# Heterocyclic Compounds: Pharmacology of Pyrazole Analogs From Rational Structural Considerations

**DOI:** 10.3389/fphar.2021.666725

**Published:** 2021-05-10

**Authors:** Rafael Fernades Costa, Larissa Córdova Turones, Keilah Valéria Naves Cavalcante, Ismael Aureliano Rosa Júnior, Carlos Henrique Xavier, Lucimar Pinheiro Rosseto, Hamilton Barbosa Napolitano, Patrícia Ferreira da Silva Castro, Marcos Luiz Ferreira Neto, Gustavo Mota Galvão, Ricardo Menegatti, Gustavo Rodrigues Pedrino, Elson Alves Costa, José Luis Rodrigues Martins, James Oluwagbamigbe Fajemiroye

**Affiliations:** ^1^ Universitary Center of Anápolis, UniEvangélica, Anápolis, Brazil; ^2^ Laboratory of Pharmacology of Natural and Synthetic Products, Institute of Biological Sciences, Federal University of Goiás, Goiânia, Brazil; ^3^ Center for Neuroscience and Cardiovascular Research, Department of Physiology, Institute of Biological Sciences, Federal University of Goiás, Goiânia, Brazil; ^4^ Institute of Science, Technology and Quality (ICTQ), Anápolis, Brazil; ^5^ Systems Neurobiology Laboratory, Department of Physiology, Institute of Biological Sciences, Federal University of Goiás, Goiânia, Brazil; ^6^ Theoretical and Structural Chemistry Group, Universidade Estadual de Goiás, Anápolis, Brazil; ^7^ Goiás State University, Itumbiara, Brazil; ^8^ Laboratory of Electrophysiology and Cardiovascular Physiology, Departament of Physiology, Institute of Biomedical Science, Federal University of Uberlândia, Uberlândia, Brazil; ^9^ Laboratory of Medicinal Pharmaceutical Chemistry, Faculty of Pharmacy, Federal University of Goiás, Goiânia, Brazil

**Keywords:** heterocyclic ring, functional groups, derivatives, interactions, activities

## Abstract

Low quality of life and life-threatening conditions often demand pharmacological screening of lead compounds. A spectrum of pharmacological activities has been attributed to pyrazole analogs. The substitution, replacement, or removal of functional groups on a pyrazole ring appears consistent with diverse molecular interactions, efficacy, and potency of these analogs. This mini-review explores cytotoxic, cytoprotective, antinociceptive, anti-inflammatory, and antidepressant activities of some pyrazole analogs to advance structure-related pharmacological profiles and rational design of new analogs. Numerous interactions of these derivatives at their targets could impact future research considerations and prospects while offering opportunities for optimizing therapeutic activity with fewer adverse effects.

## Introduction

**FIGURE 1:**
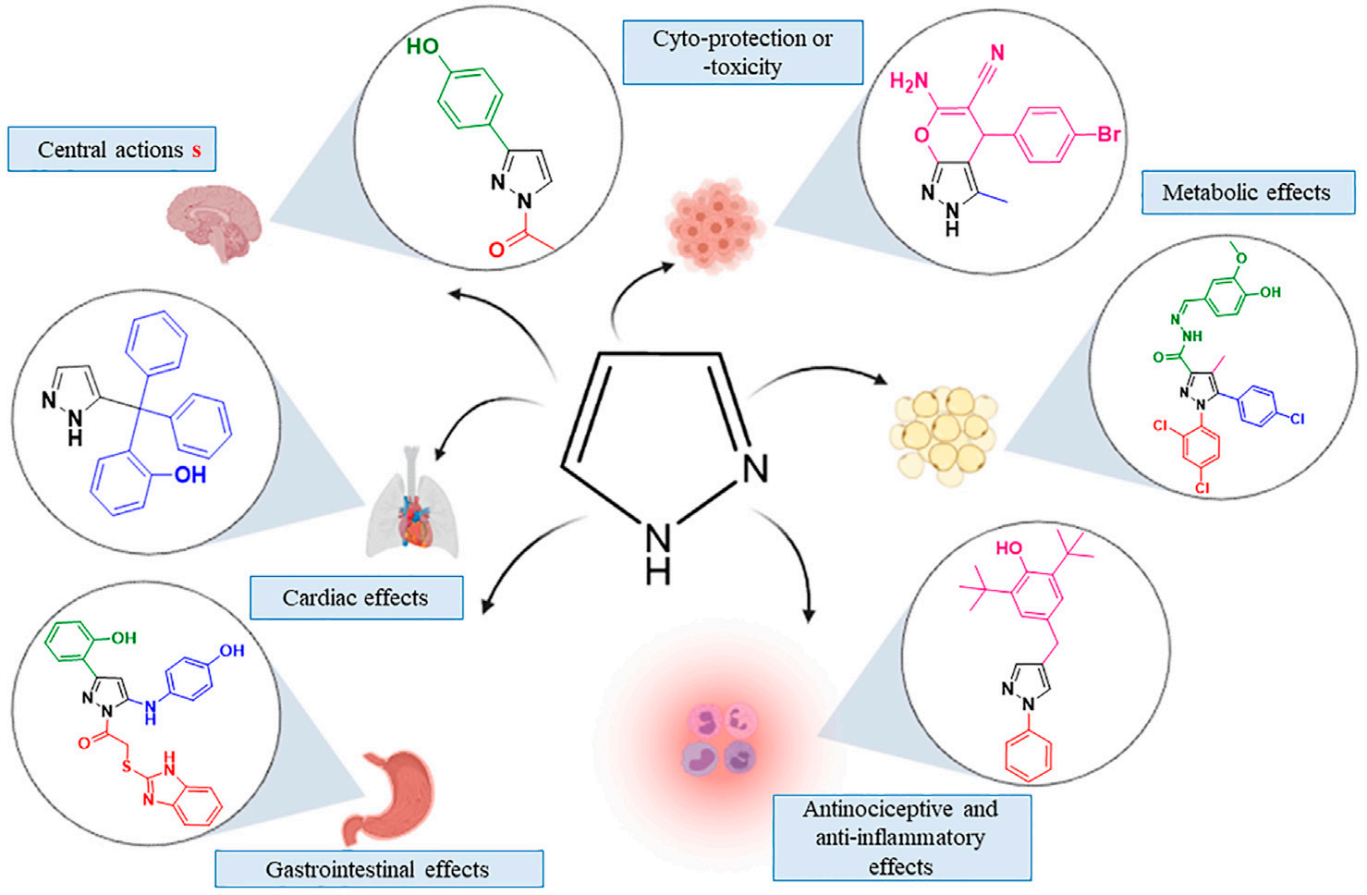
Effects evoked by pyrazole analogs in different cells target.

Medicinal chemistry is engaged in the research area to ameliorate new derivatives (Zhao, 2007). With wide application in medicine and industry (Al-Omar, 2010), the pyrazole ring is important in rational drug development. As a privileged structure present in different classes of drugs, the pyrazole moiety has inspired new classes of drug development (Faria et al., 2017; Patil, 2020; Yet, 2018). The pyrazole moiety is a nitrogen-containing heterocyclic core with diverse targets and effects (Figure 1) (Faisal et al., 2019; Ran et al., 2019; Taher et al., 2019; Badavath and Jayaprakash, 2020). The series of available pyrazole analogs could provide clues to structural activity relationship (SAR) and predict potential therapeutic or adverse effects.

**FIGURE 2:**
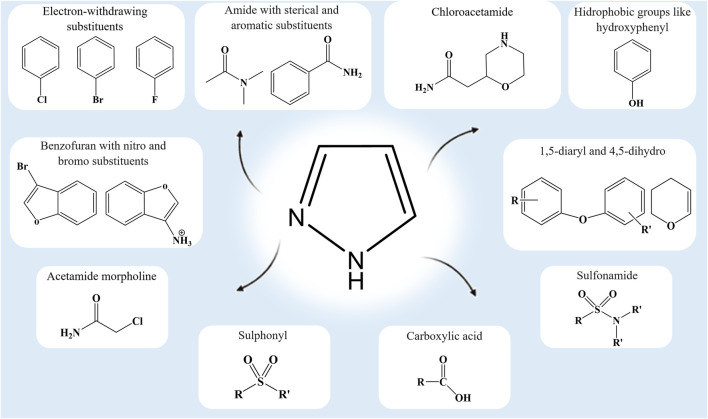
Functional groups that confer some distinctive interactions and activities on pyrazole analogs.

The substitutions, additions, removal, or fusion of different functional groups in the pyrazole ring are key to the synthesis of lead compounds that are effective against emerging and complex diseases (Faisal et al., 2019; Aziz et al., 2020; Ramsay et al., 2018; Benek et al., 2020; He et al., 2016). Pharmacological characterization of these analogs will benefit from our comprehension of the functional group modifications on the central pyrazole ring (Figure 2). The standard drugs containing the pyrazole ring [pyrazofurin (anticancer), crizotinib (cytoprotective), celecoxib and lonazolac (anti-inflammatory), difenamizole (analgesic), rimonabant (anti-obesity), sildenafil (vasodilator), and fezolamide (antidepressant)] (Hill and Whelan, 1980; Kameyama et al., 1981; Clemett and Goa, 2000; Samat et al., 2008; Straube, 2012; Dopp et al., 2013; Mitou et al., 2015; Karrouchi et al., 2018) provide ample opportunity for continuous research and analysis of new analogs.

This mini-review explores selected activities and structural modifications of some pyrazole analogs. The available data on the concentration of analogs required for 50% inhibition (IC_50_), equilibrium dissociation constant for the inhibitor (*K*_*i*_), the dose that elicited 50% of the maximum possible effect (ED_50_), ulcerogenic index (UI), or selectivity index (SI) are provided to further comparative discussions on their affinity, efficacy, and potency. As the present review is limited to selected biological activities, the author’s considerations, and future perspectives on some representative analogs (see Supplementary Table S1), additional references are listed for the aspects that are out of the present scope. All the chemical structures and figures were designed using either ChemDraw JS program or biorender.com.

## Selected Activities

### Cytotoxic and Cytoprotective Activities

The cytotoxic effects of some pyrazole analogs against specific cell lines including human breast cancer cells (MDA-MB-231 and MCF-7) appear promising to the development of anticancer drugs (Kamel, 2015). Compound **1** containing a distal pyrazole ring and sulphonyl moiety decreased the viability of MCF7 (IC_50_ = 39.70 µM). Unlike non-heterocyclic compound **4**, the activities of caspase-3 and caspase-7 in MDA-MB-231 were gradually reduced by compounds **1**–**3**. With the data showing the cleavage of caspase-8 and caspase-9 through caspase-3 and caspase-7 activation in the cascade of caspases (Toton et al., 2013), compound **4** is unlikely to elicit a specific effect. Since caspases play different roles in cellular survival, differentiation, and proliferation process, specific caspase inhibition by compounds **1**–**3** suggests a safety profile (Lehmann et al., 2017). Potent antiproliferative activity (IC_50_ = 0.26 μM) and mitogen-activated protein kinase inhibition (MEK, IC_50_ = 91 nM) have been attributed to methyl and *ortho*-fluorine groups on pyrazole carboxamide of compound **5**. This result suggests suppression of abnormal signaling of the MEK-dependent pathways. In the docking assay, the contribution of the 1,3-diphenyl-1*H*-pyrazole to hydrophobic interactions (Val 82, Ala 95, Val 127, Met 143, Met 143, and Leu 197 of the ATP-binding pocket) and MEK inhibition (Lv et al., 2016) was considered significant. Recently, a thiazole moiety was attached to the pyrazole ring and assayed against different cancer cell lines (Masaret, 2021). Relatively high cytotoxic efficacy of compounds **6**–**8** (IC_50_ = 14.32, 11.17, and 10.21, respectively) against MCF-7 supports the antitumor role of the thiazole moiety (Masaret, 2021). However, the specificity of the cytotoxic potential and emerging resistance still need to be extensively evaluated.

As some analogs are considered potent IC_50_ < 100 nM; mild IC_50_ > 10,000 nM cytotoxic or noncytotoxic on some cell lines, and others elicit cyto- or neuroprotection. The restoration of redox homeostasis or prevention of oxidative stress, inflammation, glycation, and vascular injury is key to neuroprotection (Hrelia et al., 2020). Chemically diverse pyrazole analogs have shown promising cellular or neuronal protections. The bulky dicyclohexylamide (**9**), smaller dimethylamide (**10**), and aminophenyl (**11**) (pyrazole analogs with sterically hindered substituents on amide) attenuated microglia-mediated neurotoxicity (IC_50_ = 10–50 µM) better than compound **12** (IC_50_ = 100 µM) with high susceptibility to electrophilic displacement (McKenzie et al., 2019). This compound contains an allyl group on an electron-deficient amide. Suppression of toxin-induced microglia overactivation offers a therapeutic opportunity capable of halting the progression of reactive oxygen species–induced neurodegenerative disease (Block et al., 2007). The electron-withdrawing substituents such as *para*-bromo on the aldehyde aromatic ring (**13**) elicited higher neuroprotective activity than the electron-donating substituent (Gameiro et al., 2017). This activity involves the inhibition of glycogen synthase kinase 3β (GSK3β; IC_50_ = 3.77 µM) and induction of nuclear factor (erythroid-derived 2)–like 2 (Nrf2; luciferase activity = 3–30 µM). As regulators of cellular responses, both GSK3β and Nrf2 are key neurodegenerative targets (Brandes and Gray, 2020; Toral-Rios et al., 2020).

Analogs with the *para*-bromophenyl radical attached to the pyrazole ring (**14**, **15**, and **16**) target the metabolic enzymes (acetylcholinesterase—AChE, carbonic anhydrase—hCA, and α-glycosidase—α-GlyIs) that are relevant to neurodegenerative disorders. However, the number of phenyl substituents on the pyrazole ring seems to create a specific pattern of metabolic enzyme inhibition. Turkan et al. (2018) attributed potent AChE inhibition to compound **15** with triphenyl substitutions (IC_50_ = 66.37 nM), α-GlyIs inhibition to analogs with diphenyl substitutions (**14** and **16**; IC_50_ = 43.72 and 36.02 nM, respectively), and hCA I and II isoform inhibition (IC_50_ = 0.93 and 0.75 nM, respectively) to compound **9** with two bromophenyl substitutions. Altogether, the inhibitory effects of these analogs on specific regulators of cellular processes could alter ATP production, functions of organelles, redox reactions, and proliferative, vascular, and inflammatory responses.

### Antinociceptive and Anti-inflammatory Activities

As pain is a hallmark of tissue damage and inflammatory processes (Baral et al., 2019), pyrazole analogs with antinociceptive and anti-inflammatory activities are important to analgesic drug development. The pyrazole compounds with fluorine at *para* (**17**), *meta* (**18**), and *ortho* (**19**) positions on the phenyl ring demonstrated an antinociceptive effect in the previous studies (de Oliveira et al., 2017; Florentino et al., 2019). This effect was associated with the activation of the opioid receptor and blockage of the acid-sensing ion channel subtype 1α (ASIC-1α). The *para* substitution improved the interaction with peripheral opioid receptors, while *ortho* substitution reduced the ASIC-1α channel’s affinity. The antagonism of transient receptor potential vanilloid subtype 1 (TRPV-1) seems to be optimized by the 3-chlorophenyl and 3-chloro-4-fluorophenyl substitutions (**20** and **21**, respectively) at the N1 position of the pyrazole C-region. The opioid receptors ASIC-1α and TRPV-1 are important targets of nociceptive modulation. Higher antinociceptive efficacy was reported in compound **21** (ED_50_ = 57 mg/kg). Like indomethacin (24% of pain inhibition), compound **22** with chloro and trifluoromethyl groups on phenyl and benzofuran template, respectively, elicited a similar 24% of pain inhibition (El Shehry et al., 2019). According to Kenchappa and Bodke (2020), compound **23** (benzofuran pyrazole substituted with nitro and bromo groups) inhibited pain response (60%) better than compounds **24** and **25** with benzofuran carbaldehyde (35 and 50%, respectively). These results support the elevation of antinociceptive efficacy through the addition of electron-withdrawing groups. Meanwhile, different group substitutions on other positions of pyrazole rings could offer additional data on potency and efficacy. The 4-(arylchalcogenyl)-1*H*-pyrazole analog containing the selanyl or sulfenyl group (**26** and **27**) elicited a higher nociceptive threshold in formalin, glutamate, and acid-induced abdominal writhing than compound **28** without the organochalcogen group (Oliveira et al., 2020). The modulation of oxidative and inflammatory pathways has been associated with pain inhibition (23%) by analogs with 2,3-di-tert-butylphenol (**29**) in Freund’s complete adjuvant–induced mechanical hyperalgesia (Galvão et al., 2020). A longer carbon chain (**30**) and levulinic (**31**) analogs with 47 and 50% pain inhibition, respectively (Taher et al., 2019), suggests that the length of the aliphatic chain could elicit a subtle change in the antinociceptive effect. In respect of first-line analgesic drugs, the fusion of pyrimidine moiety to the pyrazole backbone (**32**) similarly increased thermal latency (160%) and reduced abdominal writhing (83%) as compared with tramadol (175%) and aspirin (78%), respectively (Khalifa et al., 2019).

The addition of the adamantyl residue to 1,5-diaryl pyrazole (**33**) elicited a higher anti-inflammatory activity over the antinociceptive effect. The anti-inflammatory effect of pyrazole analogs could be assessed through edema formation (a cardinal sign of inflammation). Compound **33** induced a lower antiedematogenic effect than the reference drug celecoxib (39 and 82% of edema inhibition, respectively). Celecoxib exhibited higher potency of cyclooxygenase (COX)-2 inhibition than compound **33** (IC_50_ = 0.95 and 2.52 µM, respectively) (Abdelazeem et al., 2020). New azomethine compounds with an electron-withdrawing group like nitrogen (**34**) or chlorine (**35**) at the *ortho* position of phenyl ring with a satisfactory anti-inflammatory effect (ED_50_ = 0.86 and 0.92 mmol/kg, respectively) elicited weak COX-2 inhibition (IC_50_ = 38.12 and 32.11) as compared with celecoxib (ED_50_ = 8.03 mmol/kg; IC_50_ = 0.34) (Murahari et al., 2019). These results support the importance of electron-withdrawing groups toward the development of highly potent anti-inflammatory analogs. The thiohydantoin derivatives with pyrazole core and methoxy substituents (**36**–**39**) induced promising antiedematogenic activity (ED_50_ = 55–62 μmol/kg) as compared with celecoxib (ED_50_ = 78 μmol/kg). The methoxy moiety (the electron-donating group) of these analogs confers additional hydrogen bonding and interaction with COX-2 active sites (Lys68, Tyr108, Tyr341, Arg106, and Arg499). This attribute supports a higher range of binding energy among compounds **36**–**39** (16–20 kcal/mol) as compared with celecoxib (17 kcal/mol) with fewer hydrogen bonding (Ser516 and Tyr371) (Abdellatif et al., 2019). An additional evaluation revealed a better COX-2 inhibition with chloroacetamide (**40**) and acetamide morpholine (**41**) compounds (IC_50_ = 20 and 34 nM, respectively). In comparison with compound **40**, the propionamide morpholine (**42**) analog improved the selectivity index (SI = 5 and 22, respectively). Compounds **40** and **42** significantly inhibited cytosolic (31 and 44%) and microsomal (81 and 74%) prostaglandin E_2_ synthase. The antiedematogenic activity of these analogs (46 and 44%) appears similar to the analog of celecoxib (42%).

Molecular docking data portraying compound **42** as the most active analog (Hassan et al., 2019) provide clues to the role of the morpholine (heterocyclic nucleus) with electron-withdrawing substituents on the phenyl ring. Additional COX-2 interactions (Gln192 and Phe518) and improved anti-inflammatory effects seem to be related to this chemical modification. Meanwhile, the COX-2 selectivity index (SI = 417) and the anti-inflammatory effect (% edema inhibition = 87%) of compound **43** with amino and methanesulphonyl groups are very close to that of celecoxib (SI = 327; % edema inhibition = 83%). This finding suggests robust aminosulfonyl moiety interaction with Gln178, Arg499, Phe504, and Gln178 amino acid residues of COX-2 (Abdellatif et al., 2020). The growing interest in pyrazole analogs with dual COX/LOX (lipoxygenase) inhibition has widened the scope of developing potent anti-inflammatory drugs. According to Gedawy et al. (2020), analogs with benzotiophenyl and carboxylic acid (**44**) inhibited COX-2 better than celecoxib (IC_50_ = 0.01 and 0.70 µM, respectively) with almost similar 5-LOX inhibition as compared to the reference drug licofelone (IC_50_ = 1.78 and 0.51 µM, respectively). Prodrugs of compound **44** (**45** and **46** with % edema inhibition = 57 and 72%, respectively) with dual COX/LOX inhibition elicited a better anti-inflammatory effect than celecoxib (36%). Additional interactions of these analogs with COX-2 (Arg106 and Ser339) and 5-LOX (Leu368, Leu414, Ile415, and Phe421) could inspire additional modification in the subsequent series of pyrazole analogs with the anti-inflammatory potential.

### Antidepressant Activity

The level of monoamine is etiologically relevant to several psychiatric diseases such as anxiety and depression (Özdemir et al., 2020). In principle, the inhibition of monoamine oxidase (MAO) provides a good assessment of the antidepressant property of new drug candidates (Fajemiroye et al., 2015). This flavin adenine dinucleotide (FAD)–dependent enzyme exists in two isoforms (MAO-A and MAO-B). The monoamines (serotonin and norepinephrine) often associated with the etiology of depression are principal substrates of MAO-A (Özdemir et al., 2020). Analogs with a positively charged pyrazole moiety at N1 (hydroxy or dihydroxy, phenyl, chloro, methoxy, or dimethoxy groups) showed higher selectivity to MAO-A than MAO-B. The 4,5-dihydro-1*H*-pyrazoles (**47** and **48**) showed a considerable affinity with MAO-A amino acid residues (Ala68, Tyr69, Phe208, Tyr407, and Tyr444). The N1-benzenesulfonyl ring at the pyrazoline nucleus seems to mediate additional interactions with the side chain and backbone residues of amino acids at the binding pocket of MAO-A (Tripathi et al., 2018). The analogs with halogen groups in the phenyl ring could also promote hydrophobic interactions with MAO-A. The replacement of a polar 4-hydroxyphenyl substituent in pyrazoline nucleus (**47**) for a bulky hydrophobic 1-naphthyl substituent (**48**) significantly reduced the antidepressant effect. The addition of the N1-acetyl group at the pyrazole nucleus in combination with polar substituents (**49**–**53**) appears to stabilize FAD bonding and improve efficacy. According to Chimenti et al. (2006), the presence of dimethoxyphenyl on the pyrazole ring lowered the MAO inhibition potency of compound **51** (IC_50_ = 1.0 × 10^–7^ M) as compared with hydroxyphenyl radical in compound **52** (IC_50_ = 8.8 × 10^–9^ M). The incorporation of functional groups capable of increasing strain energy and facilitating ring-opening could increase the potency drastically. In this manner, the 4,5-dihydro-1*H*-pyrazole appears to be a better template for the design of MAO-A inhibitor (Chimenti et al., 2004; Tripathi et al., 2016; Upadhyay et al., 2017) than MAO-B inhibitor (Manna et al., 2002). The tautomeric forms of pyrazole by the active site of the enzyme play a key role in the development of antidepressant drugs (Secci et al., 2011). Altogether, many analogs are still without data on potency, efficacy, and MAO-A/MAO-B selectivity index that allow for comparative analysis and determination of the rational template for new pyrazole analogs with the antidepressant property. Although MAO inhibition offers a viable antidepressant mechanism, further screening of these analogs in the models of obsessive-compulsive, panic, anxiety disorder, and post-traumatic stress disorders could expand their potential applications, contraindications, and adverse events in the psychiatry setting.

### Other Activities

The modulation of central and peripheral targets by the pyrazole ring and aforementioned moieties may induce diverse autonomic manifestations. Alteration in the activity of the autonomic nervous system could impact cardiac, vascular, and respiratory functions. The pyrazole-induced endothelium-dependent vascular relaxation, sympathoinhibition, and suppression of bronchial remodeling suggest cardiovascular or respiratory effects (Girodet et al., 2013; Fajemiroye et al., 2014; Basu et al., 2017; Menegatti et al., 2019). Compounds **54** and **55** with antihypertensive properties possess a piperazine ring with carboxylic acid or phenyl substituents, respectively. The introduction of 3,5-bis(trifluoromethyl) backbone with electron-withdrawing substituents in compounds **56** (37-fold Orai1 pore inhibition potency) and **57** (18-fold transient receptor potential canonical type 3 suppression potency) reduced respiratory impairments (Schleifer et al., 2012). Bronchial remodeling suppression with details on the efficacy, potency, selectivity, or mechanism of action has been reported for calcium-activated potassium channel blocker (**58**) and adenosine receptor subtype 2_A_ inhibitor (**59**) with chloro or a *meta*-trifluoromethoxy substituent on the phenyl ring, respectively (Girodet et al., 2013; Basu et al., 2017).

Some of the analogs that share the presence of electron-withdrawing groups in common tend to interact with inflammatory mediators that are relevant to gastrointestinal and metabolic disorders. Some of these analogs inhibit COX enzymes and suppress the synthesis of prostaglandin. Although both COX-1 and COX-2 isoforms are involved in homeostatic functions, the gastrointestinal ulceration of nonsteroidal anti-inflammatory drugs (NSAIDs) has been attributed to COX-1 inhibition and subsequent reduction in gastroprotective prostaglandin (Kanno and Moayyedi, 2020). Hence, pyrazole analogs with little or no effect on prostaglandin synthesis and release remain a viable therapeutic option in patients that are highly susceptible to NSAID-induced ulceration. The anti-inflammatory and antiedematogenic pyrazole analog (**60**; 40% of edema inhibition) with a very low ulcerogenic index (UI = 60) as compared with phenylbutazone and indomethacin (UI = 275 and 300, respectively) appears promising (Maggio et al., 2001). Unlike compound **60**, both phenylbutazone and indomethacin are traditional NSAIDs well known for reducing prostaglandin levels through reversible and nonselective COX inhibition. In the ethanol-induced ulcer model, the benzimidazole–pyrazole hybrids (**61**–**66**) with substitutions on both aromatic rings of the pyrazole moiety reduced the ulcer index (UI = 72–83). The *ortho*-hydroxyl group on the phenyl ring seems to be involved in hydrogen bonding with the proton bomb (H^+^/K^+^ ATPase) since the highest binding affinity (–9.8 kcal/mole) reported for compound **64** was lost in compounds **63** and **66** with methoxy group replacement (Noor et al., 2017). The suppression of harmful gastrointestinal effects has been partially attributed to the *meta*-methyl substitution on the pyrazole ring. The mepirizole analog (**67**) with methoxy and methyl groups at *meta* and *para*-positions on a pyrimidine ring exhibited 93% gastroprotective activity. However, the replacement of the pyrazole ring of mepirizole considerably attenuated this effect (7% inhibition of ulcer formation) (Ikeda et al., 1996). These data indicate that appropriate substitutions on the pyrazole ring could reinforce gastroprotection.

The analogs that suppress pro-inflammatory mediators could also modulate insulin sensitivity and protect against metabolic syndrome. Insulin resistance, oxidative products, and atherogenic dyslipidemia are among metabolic abnormalities underlying diabetes. A molecular hybrid of rimonabant (**68**) with chloro and methoxy substitutions on the phenyl ring elicited a significant antidiabetic effect (Hernández-Vázquez et al., 2015; Hernández-Vázquez et al., 2017). In a separate study, the water-soluble pyrazole curcumin analog (**69**) inhibited advanced glycation end products (AGEs) and eliminated excess glucose (Sribalan et al., 2017). The AGEs, oxidative derivatives resulting from diabetic hyperglycemia, are increasingly seen as a potential risk for islet β-cell injury, insulin resistance, and diabetes (Vlassara and Uribarri, 2014). Although, compound **69** seems to inhibit AGEs (IC_50_ = 56.24 μg/ml) better than the parent curcumin (IC_50_ = 79.34 μg/ml) and standard drug phloroglucinol (IC_50_ = 135.73 μg/ml) sufficient data for comparative analysis of these analogs is still lacking. The chronic nature of the metabolic syndrome that warrants prolonged and often indefinite medications (Rochlani et al., 2017) makes the development of new analogs a promising strategy against the burden of adverse effects and patient nonadherence.

## Final Considerations and Future Perspective

The hunt for new analogs with desirable pharmacological profiles is a never-ending task in drug discovery programs. With the available literature on pyrazole analogs (Fadaly et al., 2020; Mohamed et al., 2020; Azimi et al., 2021; Faudzi et al., 2021; Verma et al., 2021), it is relatively easy for medicinal chemists to proceed with rational synthesis or modification capable of enhancing biological activities. The substitutions, additions, or removal of functional groups are effective strategies for designing biologically important analogs. The presence of new chemical entities could lead to additional or loss of molecular interactions. Cellular proliferation and metabolic enzymes provide important cytotoxic, cytoprotective, antinociceptive, anti-inflammatory, and antidepressant targets. However, specific contributions of the pyrazole ring and functional groups to these biological activities remain largely unclear.

In light of this review, promising cytotoxic activities of some analogs against breast cancer cell lines (MCF-7 and MDA-MB-231) is expected to stimulate extensive evaluation of their effects on lung cancer (A-549), liver cancer (HepG-2), and brain cancer (HeLa) cell lines (Lv et al., 2016; Lehmann et al., 2017; Turkan et al., 2018; McKenzie et al., 2019; Bennani et al., 2020; Masaret, 2021). As a life-threatening health issue and one of the most lethal diseases known, cancer can be managed by targeting gene expression, certain protein synthesis, functions of organelles, pH, and electrolytes (Johnston and Strobel, 2020). For instance, the cytotoxic hypothesis appears to favor exposure to halogenated analogs capable of increasing halogen influx. This small-sized functional group with high electronegativity could lower cytosolic pH (cellular acidification). According to Johnston and Strobel (2020), increasing acidification and electrolyte imbalance by fluoride could disrupt metabolic processes and induce stress signaling, underpinning cellular toxicity. However, a detailed investigation of the level of analog-induced changes in redox reactions, oxidative stress, inflammation, glycation, and other metabolic processes could facilitate the repositioning of cytotoxic analogs for cytoprotection, and vice versa. Altogether, these effects may impact autonomic and central components vis-à-vis cardiovascular, cardiopulmonary, metabolic, and affective manifestations.

Since antiproliferative and anti-inflammatory properties could reduce cellular infiltration and modulate gastrointestinal aggressive factors underlying peptic ulcer complications, in hypothesis, some pyrazole analogs could attenuate gastric ulceration, or in association with NSAIDs at subtherapeutic doses without loss of efficacy. The growing number of synthesized analogs without data of biological activity, mechanism of action, and comparative study with standard drugs containing the pyrazole ring (Hill and Whelan, 1980; Kameyama et al., 1981; Clemett and Goa, 2000; Samat et al., 2008; Straube, 2012; Dopp et al., 2013; Mitou et al., 2015; Karrouchi et al., 2018) may yield promising results, if evaluated in the future research.

As the pyrazole moiety offers a central motif to different functional groups (Basu et al., 2017; Noor et al., 2017; Tripathi et al., 2018; Murahari et al., 2019; Kenchappa and Bodke, 2020), its simplified synthetic routes and potential activities are expected to continue to inspire additional chemical modifications (Johnston and Strobel, 2020) toward clinical applications. Emerging analogs with unique physicochemical, pharmacokinetic, and pharmacodynamic properties could be useful scaffolds for future studies. The aforementioned target interactions revealed how structural modification could enrich mechanistic studies. In the future, toxicological studies, reversibility, and selectivity of the effect of these analogs as well as the potential pharmacokinetic and pharmacodynamic interactions with other drugs are expected to predict adverse and therapeutic effects.
