# Supplementary material for: Heterocyclic Compounds: Pharmacology of Pyrazole Analogs From Rational Structural Considerations
Source: Front Pharmacol. 2021 May 10;12:666725. doi: 10.3389/fphar.2021.666725 (PMC8141747; doi:10.3389/fphar.2021.666725)
Supplement: Supplementary file 1 [file DataSheet1.docx]

Supplementary Material

**Supplementary table 1.** Representative pyrazole analogs and associated functional groups.

| **Compounds** | **References** |
| --- | --- |
| *5-(1H-pyrazol-1-yl)-3-tosyl-1H-indazole* (**1**)    *5-(3,5-dimethyl-1H-pyrazol-1-yl)-3-tosyl-1H-indazole* (**2**)    *1-methyl-6-tosyl-1,4-dihydropyrazolo[4,3-c]pyrazole* (**3**)    5-chloro-3-tosyl-1*H*-indazole (**4**)  ** | Lehmann *et al.*, 2017 |
| *N-((2-fluorobenzyl)oxy)-3-phenyl-1-(p-tolyl)-1H-pyrazole4-carboxamide* (**5**)  ** | Lv *et al.*, 2016 |
| *(E)-2-(2-(4-methyl-5-(1H-pyrazol-3-yl)thiazol-2-yl)hydrazono)acenaphthylen-1(2H)-one* (**6**)  **  *(E)-2-(2-(4-methyl-5-(1-methyl-1H-pyrazol-3-yl)thiazol-2-yl)hydrazono)acenaphthylen-1(2H)-one* (**7**)    *(E)-2-(2-(4-methyl-5-(1-phenyl-1H-pyrazol-3-yl)thiazol-2-yl)hydrazono)acenaphthylen-1(2H)-one* (**8**)   | Masaret, 2021 |
| *2-Dicyclohexylamino-2-oxo-N-(4'-cyano-1'-phenyl-1H-pyrazol-5'-yl)acetamide* (**9**)    *2-(Dimethylamino)-2-oxo-N-(4'-cyano-1'-phenyl-1H-pyrazol-5'-yl)acetamide* (**10**)    *2-(4''-Aminophenyl)-2-oxo-N-(4'-cyano-1'-phenyl-1H-pyrazol-5'-yl)acetamide* (**11**)    *2-Allyl-2-oxo-N-(4'-cyano-1'-phenyl-1H-pyrazol-5'-yl)acetamide* (**12**)   | McKenzie *et al.*, 2019 |
| *6-Amino-4-(4-bromophenyl)-3-methyl-2,4-dihydropyrano[2,3-c]pyrazole-5-carbonitrile* (**13**)  ** | Gameiro *et al.*, 2017 |
| *1-(4-bromophenyl)-2-(1-methyl-3,5-diphenyl-1Hpyrazol-4yl)diazene* (**14**)    *1-(4-bromophenyl)-2-(1,3,5-triphenyl-1H-pyrazol-4yl)diazene* (**15**)    *1-(4-bromophenyl)-1-(2,5-dimethylphenyl)-3,5-diphenyl-1H-pyrazol-4yl)diazene* (**16**)   | Turkan *et al.*, 2018 |
| *5-[1-(4-fluorophenyl)-1H-pyrazol-4-yl]-2H-tetrazole* (**17**)    *5-(1-(3-fluorophenyl)-1H-pyrazol-4-yl)-2H-tetrazole* (**18**)  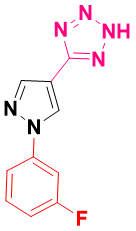  *5-(1-(2-fluorophenyl)-1H-pyrazol-4-yl)-2Htetrazole* (**19**)   | Florentino *et al.*, 2019  de Oliveira *et al.*, 2017 |
| *1-((3-(tert-butyl)-1-(3-chlorophenyl)-1H-pyrazol-5-yl)methyl)-3-(1H-indazol-4-yl)urea* (**20**)    *1-((3-(tert-butyl)-1-(3-chloro-4-fluorophenyl)-1H-pyrazol-5-yl)methyl)-3-(1H-indazol-4-yl)urea* (**21**)   | Kang *et al.*, 2020 |
| *3-[3-(4-Chlorophenyl)-1-phenyl-1H-pyrazol-4-yl]-1-[6-(trifluoromethyl)benzofuran-2-yl]prop-2-en-1-one* (**22**)   | El Shehry *et al.*, 2019 |
| *(Z)-2-((3-(5-Bromobenzofuran-2-yl)-1-(4-nitrophenyl)-1H-pyrazol-4-yl)methylene)-2, 3-dihydro-5,6-dimethoxyinden-1-one* (**23**)    *(Z)-2-((3-(5-Bromobenzofuran-2-yl)-1-(4-chlorophenyl)-1H-pyrazol-4-yl)methylene)-2,3-dihydro-5,6-dimethoxyinden-1-one* (**24**)    *(Z)-2((3-(Benzofuran-2-yl)-1-(4-nitrophenyl)-1H-pyrazol-4-yl) methylene)-2, 3-dihydro-5, 6-dimethoxyinden-1-one* (**25**)   | Kenchappa and Bodke, 2020 |
| *3,5-dimethyl-1-phenyl4-(phenylselanyl)-1H-pyrazole* (**26**)    *3,5-dimethyl-1-phenyl4-(phenylsulfenyl)-1H-pyrazole* (**27**)    *3,5-dimethyl-1-phenyl1H-pyrazole* (**28**)   | Oliveira *et al.*, 2020 |
| *2,6-di-tert-butyl-4-((1-phenyl-1H-pyrazol-4-yl)methyl)phenol* (**29**)   | Galvão *et al.*, 2020 |
| *4-Acetamidophenyl4-oxo-6-(1,3-diphenyl-1H-pyrazol-4-yl)hex-5-enoate* (**30**)   *4-Acetamidophenyl-7-(1,3-diphenyl-1H-pyrazol-4-yl)-5-oxohept6-enoate* (**31**)   | Taher *et al.*, 2019 |
| *5-(1-(3-Chlorophenyl)-3-(4-methoxyphenyl)-1H-pyrazol-4-yl)thieno[2,3-d]pyrimidin-4-amine* (**32**)   | Khalifa *et al.*, 2019 |
| *4-(3-(5-(adamantan-1-yl)-1,3,4-oxadiazol-2-yl)-5-phenyl-1H-pyrazol-1-yl)benzenesulfonamide* (**33**)  | Abdelazeem *et al.*, 2020 |
| *4-(2-nitrophenyl)-3-methyl-1-phenyl-6-[{(1e)-phenylmethylene}**amino]-1,4-dihydropyrano[2,3-c]pyrazole-5-carbonitrile* (**34**)  *4-(2-chlorophenyl)-3-methyl-1-phenyl-6-[{(1e)-**phenylmethylene}amino]-1,4-dihydropyrano[2,3-c]pyrazole-5-carbonitrile* (**35**)  | Murahari *et al.*, 2019 |
| *(Z)-3-(4-Methoxyphenyl)-5-((1-(4-(methylsulfonyl)phenyl)-3-(4-nitrophenyl)-1H-pyrazol-4-yl)methylene)-2-thioxoimidazolidin-4-one* (**36**)    *(Z)-5-((3-(4-Bromophenyl)-1-(4-(methylsulfonyl)phenyl)-1Hpyrazol-4-yl)methylene)-3-(4-methoxyphenyl)-2-thioxoimidazolidin-4-one* (**37**)    *(Z)-3-(4-Methoxyphenyl)-5-((1-(4-(methylsulfonyl)phenyl)-3-phenyl-1H-pyrazol-4-yl)methylene)-2-thioxoimidazolidin-4-one* (**38**)    *(Z)-5-((1-(4-(Methylsulfonyl)phenyl)-3-(4-nitrophenyl)-1Hpyrazol-4-yl)methylene)-3-phenyl-2-thioxoimidazolidin-4-one* (**39**)   | Abdellatif *et al.*, 2019 |
| *2-Chloro-N-[4-cyano-1-(4-sulfamoylphenyl)-1H-pyrazol-5-**yl]acetamide* (**40**)  *N-[4-cyano-1-(4-sulfamoylphenyl)-1H-pyrazol-5-yl]-2-**morpholinoacetamide* (**41**)  *N-[4-cyano-1-(4-sulfamoylphenyl)-1H-pyrazol-5-yl]-3-**morpholino-propionamide* (**42**)  | Hassan *et al.*, 2019 |
| *5-Amino-1-[5-amino-3-methyl-1-(4-sulfamoylphenyl)-1Hpyrazole-**4-carbonyl]-1H-pyrazole-4-carboxylic acid ethyl ester* (**43**) | Abdellatif *et al.*, 2020 |
| *5-(Benzo[b]thiophen-2-yl)-1-(4-sulfamoylphenyl)-1H-pyrazole-3-carboxylic acid* (**44**)    *Methyl 5-(benzo[b]thiophen-2-yl)-1-(4-sulfamoylphenyl)-1H-pyrazole-3-carboxylate* (**45**)    *Ethyl 5-(benzo[b]thiophen-2-yl)-1-(4-sulfamoylphenyl)-1Hpyrazole-3-carboxylate* (**46**)   | Gedawy *et al.*, 2020 |
| *4-[1-(4-Nitro-benzenesulfonyl)-5-thiophen-2-yl-4,5-dihydro-1H-pyrazol-3-yl]-*phenol (**47**)    *3-Naphthalen-1-yl-1-(4-nitro-benzenesulfonyl)-5-thiophen-2-yl-4,5-dihydro-1H-pyrazole* (**48**)   | Tripathi *et al.*, 2018 |
| *1-(5-(3-chlorophenyl)-3-(4-hydroxyphenyl)-1H-pyrazol-1-yl)ethan-1-one* (**49**)    *1-(3-(4-hydroxyphenyl)-5-(2-methoxyphenyl)-1H-pyrazol-1-yl)ethan-1-one* (**50**)  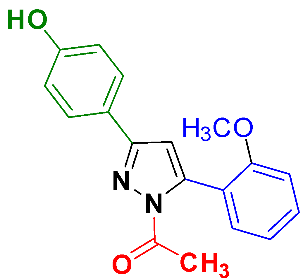  *1-(5-(3,4-dimethoxyphenyl)-3-(2-hydroxyphenyl)-1H-pyrazol-1-yl)ethan-1-one* (**51**)    *1-(3-(4-hydroxyphenyl)-1H-pyrazol-1-yl)ethan-1-one* (**52**)    *1-(3-(2,4-dihydroxyphenyl)-1H-pyrazol-1-yl)ethan-1-one* (**53**)   | Chiment *et al.*, 2006 |
| *4-[(1-phenyl-1H-pyrazol-4-yl) methyl]1-piperazine carboxylic acid ethyl ester* (**54**)  | Fajemiroye *et al.*, 2014 |
| *2-(4-(1-phenyl-1H-pyrazol-4-yl)methyl) piperazin-1-yl)etan-1-ol* (**55**) 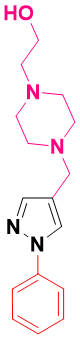 | Menegatti *et al.*, 2019 |
| *[(N-(4-(3,5-bis(trifluoromethyl)-1H-pyrazole-1-yl)phenyl)-3-fluoroisonicotinamide* (**56**) 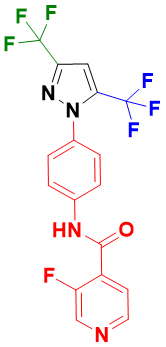  *N-(4-(3,5-bis(trifluoromethyl)-1H-pyrazole-1-yl)phenyl)-4-methylbenzenesulfonamide* (**57**)  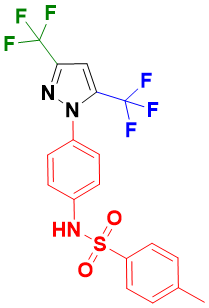 | Schleifer *et al.*, 2012 |
| *5-[(2-chlorophenyl)(diphenyl)methyl] -1H-pyrazole* (**58**) 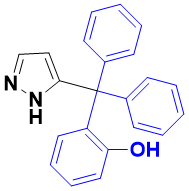 | Girodet *et al.*, 2013 |
| *3-ethyl-1-propyl-8-(1-(3-(3-(trifluoromethoxy)phenyl)prop-2-yn-1-yl)-1H-pyrazol-4-yl)-3,7-dihydro-1H-purine-2,6-dione* (**59**)  | Basu *et al.*, 2017 |
| *ethyl 2-(5-(7-chloro-4-oxo-2-phenylquinazolin-3(4H)-yl)-1-methyl-1H-pyrazol-4-yl)acetate* (**60**)  | Maggio *et al.*, 2001 |
| *2(1H.benzimidazol.2.ylsulfanyl).1.[5.(2.hydroxyphenyl).3.phenyl.1H.pyrazol.1.yl]ethanone* (**61**)    *2‑(1H‑benzimidazol‑2‑yl sulfanyl)‑1‑[3,5‑bis(2‑hydroxyphenyl)‑1H‑pyrazol‑1‑yl]ethanone* (**62**)    *2-(1H-benzimidazol-2-ylsulfanyl)-1-[5-(2-hydroxyphenyl)-3-(3-hydroxy-4-methoxyphenyl)-1H-pyrazol-1-yl]ethanone* (**63**)    *2‑(1H‑benzimidazol‑2‑ylsulfanyl)‑1‑[5‑(4‑hydroxyphenylamino)‑3‑(2‑hydroxyphenyl)‑1H‑pyrazol‑1‑yl]ethanone* (**64**)    *2-(1H-benzimidazol-2-ylsulfanyl)-1-(3,5-diphenyl-1H-pyrazol-1-yl)ethanone* (**65**)    *2‑(1H‑benzimidazol‑2‑yl-sulfanyl)‑1‑[5‑(3‑hydroxy‑4‑methoxyphenyl)‑3‑phenyl‑1H‑pyrazol‑1‑yl]ethanone* (**66**)   | Noor *et al.*, 2017 |
| *4-methoxy-6-methyl-2-(1H-pyrazol-1-yl)pyrimidine* (**67**)  | Ikeda *et al.*, 1996 |
| *[(5-(4-chlorophenyl)-1-(2,4-dichlorophenyl)-N´-[(Z)-(4-hydroxy-3-methoxyphenyl)methylidene]-4-methyl-1H-pyrazol-3-carbohydrazide* (**68**)  | Hernández-Vázquez *et al.*, 2015, 2017 |
| *sodium 2,2'-((((1E,1'E)-(1H-pyrazole-3,5-diyl)bis(ethene-2,1-diyl))bis(2-ethyl-4,1-phenylene))bis(oxy))diacetate* (**69**)  | Sribalan *et al.*, 2017 |
